# Functional redundancy of two ABC transporter proteins in mediating toxicity of *Bacillus thuringiensis* to cotton bollworm

**DOI:** 10.1371/journal.ppat.1008427

**Published:** 2020-03-19

**Authors:** Jing Wang, Huanhuan Ma, Shan Zhao, Jianlei Huang, Yihua Yang, Bruce E. Tabashnik, Yidong Wu

**Affiliations:** 1 College of Plant Protection, Nanjing Agricultural University, Nanjing, China; 2 Department of Entomology, University of Arizona, Tucson, Arizona, United States of America; University of Texas Medical School at Houston, UNITED STATES

## Abstract

Evolution of pest resistance reduces the efficacy of insecticidal proteins from the gram-positive bacterium *Bacillus thuringiensis* (Bt) used widely in sprays and transgenic crops. Better understanding of the genetic basis of resistance is needed to more effectively monitor, manage, and counter pest resistance to Bt toxins. Here we used CRISPR/Cas9 gene editing to clarify the genetics of Bt resistance and the associated effects on susceptibility to other microbial insecticides in one of the world’s most damaging pests, the cotton bollworm (*Helicoverpa armigera*). We discovered that CRISPR-mediated knockouts of ATP-binding cassette (ABC) transporter genes *HaABCC2* and *HaABCC3* together caused >15,000-fold resistance to Bt toxin Cry1Ac, whereas knocking out either *HaABCC2* or *HaABCC3* alone had little or no effect. Inheritance of resistance was autosomal and recessive. Bioassays of progeny from interstrain crosses revealed that one wild type allele of either *HaABCC2* or *HaABCC3* is sufficient to sustain substantial susceptibility to Cry1Ac. In contrast with previous results, susceptibility to two insecticides derived from bacteria other than Bt (abamectin and spinetoram), was not affected by knocking out *HaABCC2*, *HaABCC3*, or both. The results here provide the first evidence that either HaABCC2 or HaABCC3 protein is sufficient to confer substantial susceptibility to Cry1Ac. The functional redundancy of these two proteins in toxicity of Cry1Ac to *H*. *armigera* is expected to reduce the likelihood of field-evolved resistance relative to disruption of a toxic process where mutations affecting a single protein can confer resistance.

## Author summary

Insect-killing proteins from the soil bacterium *Bacillus thuringiensis* (Bt) are increasingly important as environmentally friendly alternatives to conventional insecticides. However, widespread planting for the past two decades of crops genetically engineered to produce Bt proteins has spurred rapid evolution of resistance in pests. Better understanding of the genetic basis of pest resistance to Bt toxins is urgently needed to address this problem. We used CRISPR gene editing to clarify the genetics of resistance to Bt toxins in the cotton bollworm, one of the world’s most damaging pests. We discovered that CRISPR-mediated inactivation of two larval midgut transporter proteins that act as receptors for Bt toxins caused a 15,000-fold increase in the concentration of Bt toxin needed to kill fifty per cent of larvae. By contrast, inactivating each of these transporter proteins alone had little or no effect. These results provide the first evidence that either of these two transporter proteins can confer susceptibility to Bt toxins. Because resistance to Bt toxins requires mutations in each of the two genes encoding these two proteins, pest resistance is less likely to evolve via this route relative to interference with toxic processes where a single mutation can confer resistance.

## Introduction

Insecticidal proteins from the gram-positive soil bacterium *Bacillus thuringiensis* (Bt) are used extensively in sprays and transgenic plants to control insects that devour crops and vector diseases [1,2]. These Bt proteins are especially valuable because they kill some devastating pests, but are not toxic to humans and most other non-target organisms [1,3,4]. Worldwide planting of transgenic crops that produce Bt proteins increased from 1.1 million hectares in 1996 to 104 million hectares in 2018 [5]. Benefits of Bt crops include pest suppression and reduced use of conventional insecticides [6–11]. However, rapid evolution of resistance to Bt toxins by pests has diminished these benefits [12,13]. Practical resistance, which is field-evolved resistance that has practical consequences for pest control, is documented for Bt sprays in some populations of two major pests [14,15]. Practical resistance to Bt crops has increased from three cases in 2005 to at least 22 cases affecting nine major pest species [12,13,16]. Better understanding of the genetic basis of resistance is urgently needed to more effectively monitor, manage, and counter pest resistance to Bt toxins.

The most common and most potent mechanism of insect resistance to Bt toxins is disruption of toxin binding to larval midgut receptors, particularly cadherins and ATP-binding cassette (ABC) transporter proteins [17–19]. Resistance to crystalline (Cry) toxins of Bt in the Cry1, Cry2 or Cry3 families is associated with ABC transporter proteins in some lab-selected strains and field-selected populations of at least nine insect species [19–27]. In addition to studies implicating several ABC transporter proteins in resistance to Cry toxins, extensive evidence indicates many members of the superfamily of ABC transporter proteins protect cells by excreting xenobiotics, including ABC transporters that confer resistance to drugs and chemotherapy agents in humans and resistance to insecticides other than Bt in arthropods [20,28,29]. Although the association between mutations in or down-regulation of the ABC transporter protein ABCC2 and resistance of lepidopterans to Bt toxins in the Cry1 family is well established, less is known about its paralog ABCC3 and interactions between ABCC2 and ABCC3 [30–40].

Here we used CRISPR/Cas9 editing to determine the independent and joint effects of knocking out the genes encoding ABCC2 and ABCC3 in the cotton bollworm, *Helicoverpa armigera*. This lepidopteran is one of the world’s most devastating crop pests and has recently invaded the Americas [41,42]. Although Bt cotton producing Cry1Ac remains effective against this major pest in China, many strains of this species have been selected for resistance to Cry1Ac in the laboratory and ‘‘early warning” of increases in the frequency of resistance to Cry1Ac has been reported from field populations in northern China exposed intensively to Bt cotton [43–45]. Previous work showed that 1100-fold resistance to activated Cry1Ac toxin in the lab-selected LF60 strain of this pest from China was linked with a 6-bp deletion in the gene encoding ABCC2 (*HaABCC2*) that disrupts splicing and introduces a premature stop codon [33]. This resistance was associated with increased susceptibility to two insecticides derived from soil bacteria other than Bt (abamectin from *Streptomyces avermitilis* and spinetoram from *Saccharopolyspora spinosa*, [46]). These previous results support the hypothesis that Bt resistance mutations disrupting ABC transporters interfere with the protective function of the transporters and thereby increase susceptibility to other insecticides [20,46]. However, the previous work did not examine ABCC3 and analyzed a resistant strain generated by conventional laboratory selection, which could have selected for resistance alleles at *HaABCC2* and other loci. In contrast with the previous results, we discovered that knocking out *HaABCC2* alone caused only 3.8-fold resistance to Cry1Ac, whereas knocking out both *HaABCC2* and *HaABCC3* caused >15,000-fold resistance to Cry1Ac, but did not increase susceptibility to abamectin or spinetoram.

## Results

### CRISPR/Cas9 knockouts of *HaABCC2*, *HaABCC3*, and both

**Fig 1:**
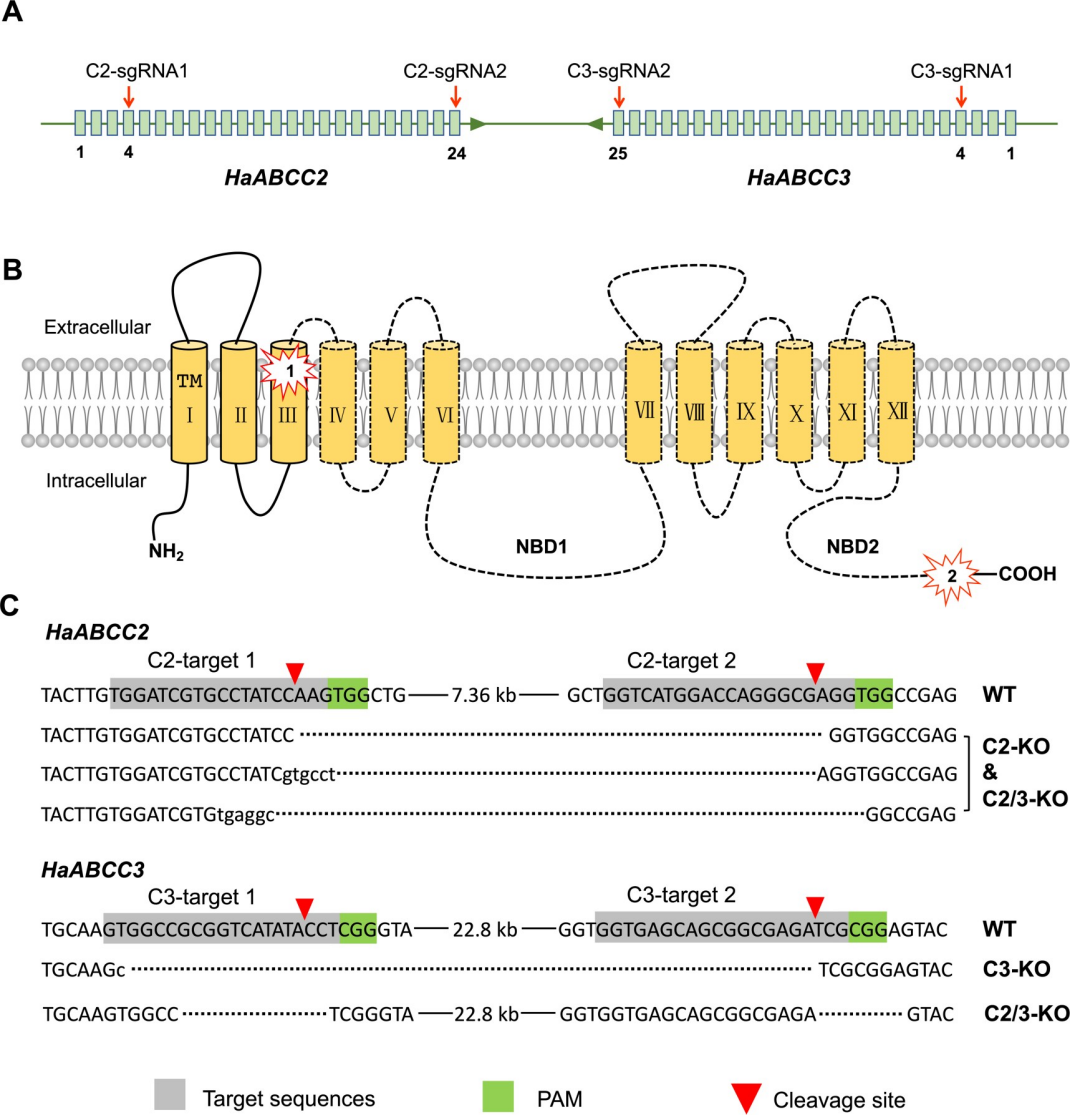
CRISPR/Cas9-mediated knockout of *HaABCC2* and *HaABCC3*. A) Genomic DNA. C2-sgRNA1 and C2-sgRNA2 target exons 4 and 24 in *HaABCC2*. C3-sgRNA1 and C3-sgRNA2 target exons 4 and 25 in the adjacent gene, *HaABCC3*. Boxes show exons and lines show introns. B) Protein structure for both HaABCC2 and HaABCC3. The number 1 shows the target sites of C2-sgRNA1 and C3-sgRNA1 in transmembrane (TM) region III; 2 shows the target sites of C2-sgRNA2 and C3-sgRNA2 near the carboxy terminus. C) Sequences of the wild type (WT) and knockouts (C2-KO, C3-KO, and C2/3-KO) detected in G_1_. Dashes show deleted bases and lower case letters show inserted bases. For *HaABCC2*, the same three knockout sequences occurred in C2-KO and C2/3-KO. For *HaABCC3*, one knockout sequence (large fragment missing) occurred in C3-KO and a different knockout sequence (two small fragments missing) occurred in C2/3-KO.

To knockout *HaABCC2* and create knockout strain C2-KO, we injected C2-sgRNA1 and C2-sgRNA2 into eggs of susceptible strain SCD and reared the resulting neonates to adults (G_0_) (Table 1). To produce G_1_ progeny, we crossed the G_0_ adults with SCD (female G_0_ X male SCD and female SCD X male G_0_). After pupation of the G_1_, we used exuviate-based PCR with specific primers (Table 2) to detect DNA fragments with knockouts in *HaABCC2*. Direct sequencing revealed three different *HaABCC2* knockout sequences from the 10 individuals analyzed (Fig 1C). Each of the three knockout sequences lacks ~7 kb between exons 4 and 24 (Fig 1C). To generate G_2_, we pooled the 10 G_1_ adults (six females and four males) for mating. After pupation of the G_2_, we used exuviate-based PCR to detect individuals with knockouts at both *HaABCC2* alleles (including those homozygous for the same knockout and those with two different knockouts), from which we established knockout strain C2-KO.

**Table 1:** Hatch rate and genotype frequencies during the creation of knockout strains.

Strain	Hatch rate (G_0_)^*a*^	Heterozygote frequency (G_1_)^*b*^	Homozygote frequency (G_2_)^*c*^
C2-KO	24.2% (116/480)	0.104 (10/96)	0.100 (16/160)
C3-KO	54.7% (173/316)	0.068 (13/192)	0.094 (9/96)
C2/3-KO	49.3% (116/235)	0.083 (8/96)	0.211 (162/768)

^a^ (Neonates / eggs injected) X 100%.

^*b*^ Heterozygotes / total number of pupae screened.

^c^ Homozygous mutants / total number of pupae and larvae screened; based on sequencing of all individuals screened for C2-KO and C3-KO, inferred from 21.1% survival at the diagnostic concentration and results showing that all 24 survivors sequenced were homozygous for the double knockout for C2/3-KO.

**Table 2:** Primers used to identify mutations at four target sites.

Target site	Primer name^a^	Primer sequences (5’>3’)	Product size
C2-target 1	C2-tar1-F	TTGCTCCGCATGACTCAAGTGTC	~210 bp
	C2-tar1-R	AAAATCAGTATAACGACTCCAAA	
C2-target 2	C2-tar2-F	CGGACGCTTTGATCCAAAAGACG	~230 bp
	C2-tar2-R	CCTCCATTAAGGTCCTTGTCATG	
C3-target 1	C3-tar1-F	TGCGTATGAGCAACGGTTCCCTG	~220 bp
	C3-tar1-R	TGGAAAGGCAGAGCAAGAGGAT	
C3-target 2	C3-tar2-F	CTGACAGCTTCATCCAGGAAACG	~270 bp
	C3-tar2-R	CTTGCGTTTTCTTTCAGATTGCT	

^a^ The primer underlined was used for direct sequencing.

We used analogous procedures to knockout *HaABCC3* and create knockout strain C3-KO. Eggs were injected with C3-sgRNA1 and C3-sgRNA2. Direct sequencing of G_1_ with exuviate-based PCR revealed only one type of *HaABCC3* knockout among the 13 individuals sequenced. This knockout lacks ~22 kb between exon 4 and 25. To generate G_2_, we pooled the 13 G_1_ adults (five females and eight males) for mating. After pupation of the G_2_, we used exuviate-based PCR to detect individuals homozygous for the single *HaABCC3* knockout, from which we began knockout strain C3-KO.

To produce strain C2/3-KO with both *HaABCC2* and *HaABCC3* knocked out, we injected C2-KO eggs with C3-sgRNA1 and C3-sgRNA2. As described above, the G_0_ adults were crossed with SCD to generate G_1_. Using exuviate-based PCR with the primers designed to detect the intended 22.8 kb deletion in *HaABCC3* (Fig 1, C3-tar1-F/C3-tar2-R, Table 2), we did not find this large deletion in any of the 96 individuals tested. However, we did find a different deletion that was shared by eight G_1_ individuals: 13 bp missing at the C3-target 1 site and 7 bp missing at the C3-target 2 site. These eight G_1_ adults (four of each sex) were pooled for mating to produce G_2_ (Fig 1C) (Table 1). Screening of 768 G_2_ larvae at the diagnostic concentration of Cry1Ac (0.05 μg Cry1Ac per cm^2^) yielded 21.1% (162/768) survival (Table 1). From the screened larvae, we used direct sequencing to determine the *HaABCC2* and *HaABCC3* genotype of 24 survivors and 24 dead individuals. All of the survivors had knockout sequences at both alleles for *HaABCC2* and *HaABCC3*, whereas none of the 24 dead larvae had knockouts at both alleles for both genes (five homozygous wild type at both loci and 19 with a knockout allele and a wild type allele at both loci). The proportion of individuals with a knockout at both alleles of both genes was significantly higher in the survivors than the dead larvae (Fisher’s exact test, P = 6 X 10^−14^), indicating strong genetic linkage between the double knockout and resistance to Cry1Ac. We pooled the 162 survivors to establish double knockout strain C2/3-KO.

Based on the frequency of heterozygotes in the G_1_ of 0.068 to 0.104 (Table 1), we infer the germline conversion rates in the G_0_ were at least approximately 7 to 10%. For the G_2_, which were generated by matings between G_1_ heterozygotes, the expected frequency of homozygous mutants is 0.25. The observed frequency of homozygous mutants for G_2_ was significantly lower than expected for the knockouts of *HaABCC2* and *HaABCC3* alone (0.100 and 0.094, respectively, Table 1; Fisher’s exact test, P < 0.01 in each case), but not for the second knockout used to generate C2/3-KO (0.211, Fisher’s exact test, P = 0.078). The significantly lower than expected frequency of homozygous mutants in G_2_ for each single knockout may reflect a fitness cost in these insects relative to insects homozygous or heterozygous for the wild-type (susceptible) allele. By contrast, in the double knockouts, all G_2_ individuals were homozygous for the C2 knockout. Thus, the lack of a significant deficit of homozygous mutants in G_2_ suggests the fitness of individuals with the double knockout was not substantially lower than the fitness of individuals with only the C2 knockout.

### Effects of knockouts on susceptibility to three Cry1A toxins

Relative to its susceptible parent strain SCD, the resistance ratio of double knockout strain C2/3-KO was >300 for Cry1Aa, >1400 for Cry1Ab, and >15,000 for Cry1Ac (Table 3). Results at the highest concentration tested of each toxin show mortality of C2/3-KO was 14.6% at 50 μg Cry1Aa per cm^2^ diet, 0% at 50 μg Cry1Ab per cm^2^ diet, and 16.7% at 80 μg Cry1Ac per cm^2^ diet (Table 3). Knockout strain C2-KO was not resistant to Cry1Aa, but had significant, 4.0-fold resistance to Cry1Ab and 3.8-fold resistance to Cry1Ac (Table 3). Knockout strain C3-KO was not resistant to any of the three Cry1A toxins (Table 3).

**Table 3:** Responses to Cry1A toxins of the susceptible SCD strain and three knockout strains.

Toxin	Strain	Slope ± SE	LC_50_ (95% FL^a^)	RR^b^
Cry1Aa	SCD	1.9 ± 0.18	0.16 (0.12–0.22)	1.0
	C2-KO	2.0 ± 0.18	0.14 (0.11–0.16)	0.9
	C3-KO	2.2 ± 0.20	0.17 (0.12–0.21)	1.1
	C2/3-KO	—	>50 ^d^	>300
Cry1Ab	SCD	2.1 ± 0.19	0.035 (0.026–0.047)	1.0
	C2-KO	1.8 ± 0.17	0.14 (0.10–0.19)	4.0 ^c^
	C3-KO	2.4 ± 0.21	0.034 (0.026–0.045)	1.0
	C2/3-KO	—	>50 ^d^	>1400
Cry1Ac	SCD	1.9 ± 0.18	0.0053 (0.0043–0.0064)	1.0
	C2-KO	1.8 ± 0.17	0.020 (0.016–0.025)	3.8 ^c^
	C3-KO	2.1 ± 0.19	0.0047 (0.0033–0.0065)	0.9
	C2/3-KO	—	>80 ^d^	>15,000

^a^ 95% fiducial limits, units are μg toxin per cm^2^ diet.

^b^ Resistance ratio = LC_50_ of strain divided by LC_50_ of SCD for the same toxin.

^c^ LC_50_ of the same toxin significantly greater for the knockout strain than SCD by the conservation criterion of no overlap of the 95% fiducial limits.

^d^ The highest concentration tested (50 or 80 μg toxin per cm^2^ diet, as indicated above) killed less than 17% of larvae.

**Fig 2:**
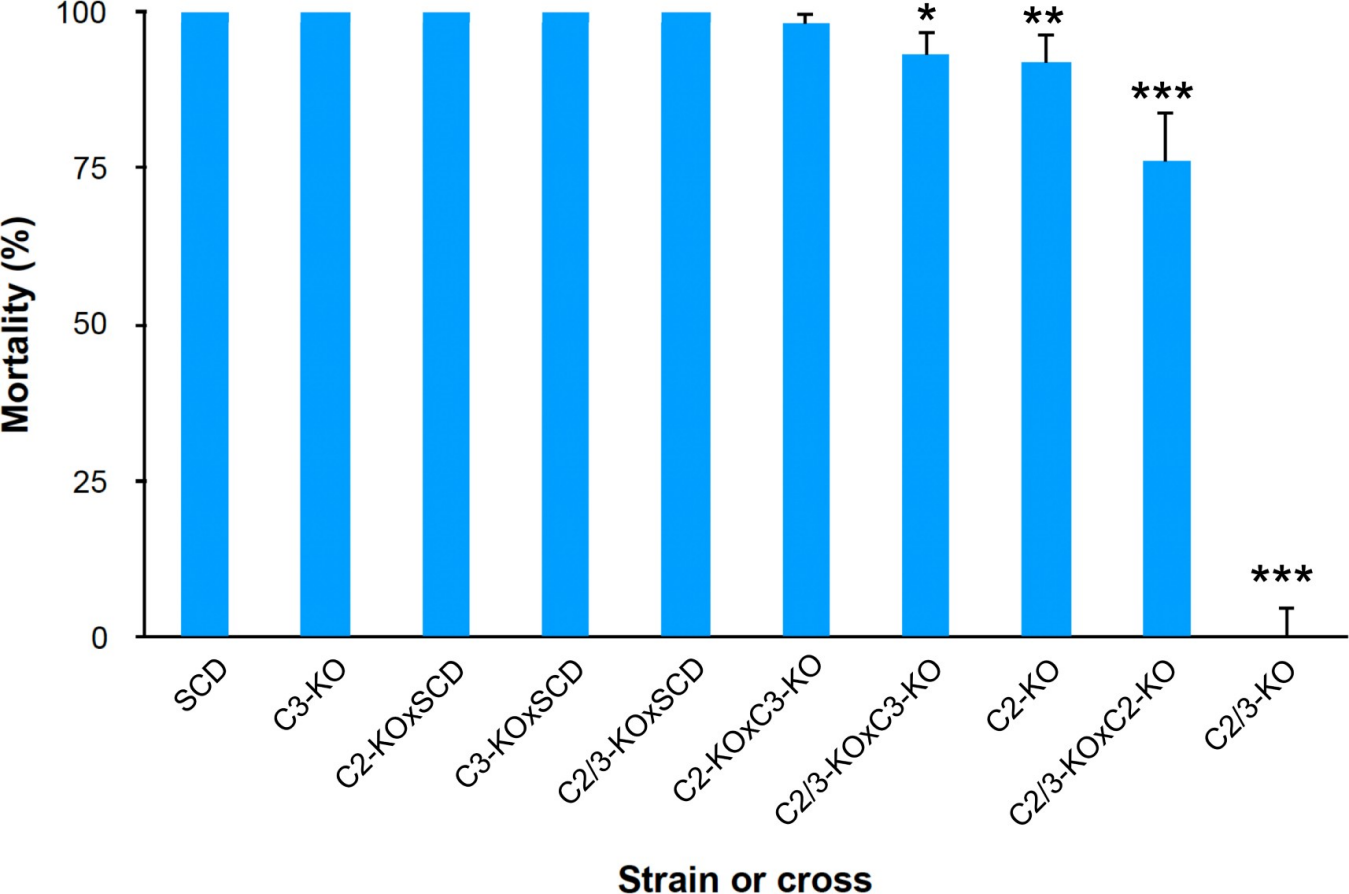
Mortality of the susceptible strain (SCD), strains with one or two knockouts (C2-KO, C3-KO, and C2/3-KO), and progeny of various crosses in bioassays with Cry1Ac (0.05 μg Cry1Ac per cm^2^ diet). Mortality was 100% for the first five strains and crosses at the left and 0% for C2/3-KO. Error bars show the upper limit of the 95% confidence interval. Asterisks indicate the two strains and two crosses with significantly lower mortality than SCD; P values from Fisher’s exact test: * = 0.014, ** = 0.0067, *** = 5 X 10^−8^ for C2/3-KO X C2-KO and 5 X 10^−57^ for C2/3-KO.

Mortality at the diagnostic concentration of Cry1Ac (0.05 μg Cry1Ac per cm^2^) was 100% for SCD and 0% for C2/3-KO (Fig 2), consistent with the high resistance ratio for the double knockout strain reported above. Also consistent with the results above, mortality at this concentration was slightly and significantly lower for C2-KO (92%) than SCD, but did not differ between C3-KO (100%) and SCD (Fig 2).

### Inheritance of resistance to Cry1Ac

We evaluated inheritance of resistance based on mortality caused by the diagnostic concentration of Cry1Ac. For the F_1_ progeny from each of the six interstrain crosses between SCD, C2/3-KO, C2-KO, and C3-KO, mortality at this concentration did not differ significantly between reciprocal crosses, indicating no sex linkage or maternal effects were evident. Because mortality did not differ significantly between the two reciprocal crosses for each interstrain cross, we pooled the results of the two reciprocal crosses for each of the six interstrain crosses. Mortality was 100% for SCD and for the F_1_ progeny from C2/3-KO X SCD and C2-KO X SCD, indicating completely recessive inheritance of resistance at the diagnostic concentration (Fig 2). Likewise, relative to SCD, mortality was not significantly lower for the F_1_ progeny from C2-KO × C3-KO (98%, Fisher’s exact test, P = 0.50), which are expected to have one knockout allele and one wild type allele at each locus. However, relative to SCD, mortality was significantly lower for the F_1_ progeny from C2/3-KO × C3-KO (93%) and C2/3-KO × C2-KO (76%) (Fisher’s exact tests, P = 0.014 and 5 X 10^−8^, respectively, Fig 2). Mortality was also significantly lower for the F_1_ progeny from C2/3-KO X C2-KO than from C2/3-KO X C3-KO (Fisher’s exact test, P = 0.0024).

### Effects of knockouts on susceptibility to three insecticides

Relative to SCD, none of the three knockout strains showed significantly different susceptibility to the insecticides abamectin, spinetoram or chlorantraniliprole (Table 4).

**Table 4:** Responses to three insecticides of the susceptible SCD strain and three knockout strains.

Insecticide	Strain	Slope ± SE	LC_50_ (95% FL^a^)	RR^b^
Abamectin	SCD	2.5 ± 0.24	0.076 (0.049–0.121)	1.0
	C2-KO	2.7 ± 0.25	0.074 (0.063–0.088)	1.0
	C3-KO	2.9 ± 0.28	0.075 (0.056–0.100)	1.0
	C2/3-KO	3.6 ± 0.38	0.073 (0.063–0.084)	1.0
Spinetoram	SCD	3.5 ± 0.34	0.25 (0.22–0.29)	1.0
	C2-KO	3.2 ± 0.30	0.24 (0.19–0.30)	1.0
	C3-KO	3.3 ± 0.31	0.25 (0.22–0.29)	1.0
	C2/3-KO	2.8 ± 0.26	0.24 (0.20–0.28)	1.0
Chlorantraniliprole	SCD	3.4 ± 0.35	0.30 (0.26–0.35)	1.0
	C2-KO	3.3 ± 0.33	0.34 (0.29–0.40)	1.1
	C3-KO	2.9 ± 0.28	0.37 (0.32–0.44)	1.2
	C2/3-KO	3.3 ± 0.33	0.31 (0.27–0.36)	1.0

^a^ 95% fiducial limits, units are mg per liter.

^b^ Resistance ratio = LC_50_ of strain divided by LC_50_ of SCD.

## Discussion

**Fig 3:**
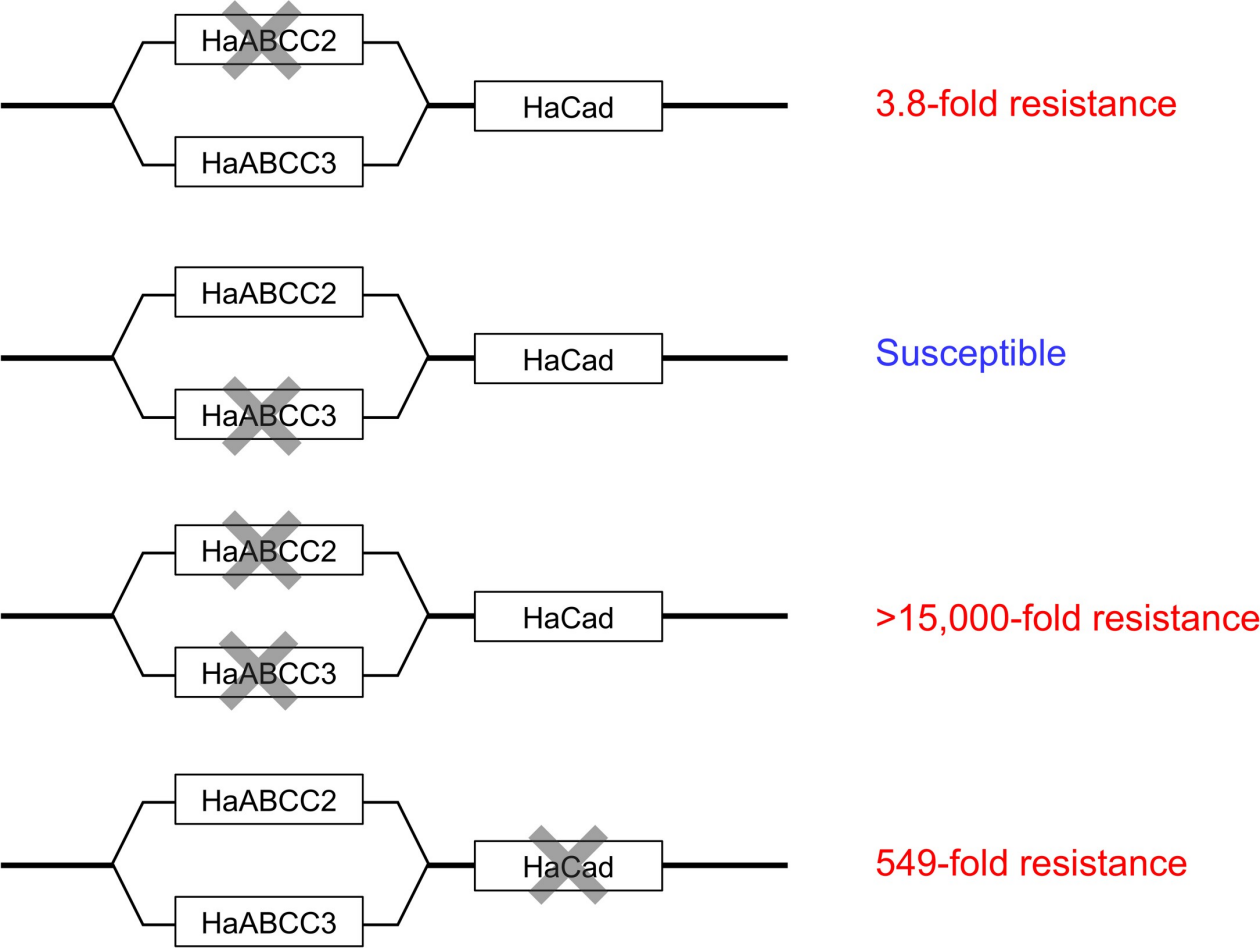
Proposed model for the toxic pathway of Cry1Ac against *H*. *armigera* based on results from CRISPR-mediated editing of *HaABCC2* and *HaABCC3* in this study, and of *HaCad* [47].

In laboratory bioassays with *H*. *armigera*, we discovered that the CRISPR-mediated double knockout of *HaABCC2* and *HaABCC3* caused >15,000-fold resistance to Cry1Ac, whereas knocking out *HaABCC2* alone caused only 3.8-fold resistance to Cry1Ac and knocking out *HaABCC3* alone did not decrease susceptibility. Likewise, at a single concentration of Cry1Ac, mortality was 0% for the double knockout strain C2/3-KO, 92% for strain C2-KO with *HaABCC2* knocked out, and 100% for strain C3-KO with *HaABCC3* knocked out. These results suggest that to a large extent, HaABCC2 and HaABCC3 act in parallel in the toxic pathway of Cry1Ac, with complete or nearly complete susceptibility retained when only one of these proteins is disrupted (Fig 3).

The extremely high resistance to Cry1Ac generated from knocking out both *HaABCC2* and *HaABCC3* implies that toxicity of Cry1Ac requires at least one of the two proteins encoded by these genes. The results also suggest that when each protein is considered alone, HaABCC2 is somewhat more important for susceptibility than HaABCC3, because knocking out *HaABCC2* alone caused low, but significant resistance, whereas knocking out *HaABCC3* alone caused none.

The results reported here from bioassays of the F_1_ progeny of various crosses show recessive inheritance of the resistance that was caused by knocking out *HaABCC2* alone or *HaABCC2* and *HaABCC3* together. Moreover, substantial susceptibility to Cry1Ac was retained in F_1_ progeny that had at least one wild type *HaABCC2* or *HaABCC3* allele. Consistent with the results from strains C2-KO and C3-KO described above, results from the F_1_ progeny show that a single wild type *HaABCC2* allele conferred greater susceptibility than a single wild type *HaABCC3* allele.

In *H*. *armigera*, the toxic pathway for Cry1Ac also requires wild type cadherin (HaCad) (Fig 3), based on previous results showing that knockout of the *HaCad* gene caused 549-fold resistance to Cry1Ac [47]. Also, the single amino acid substitution L31S in HaTSPAN1 produced 125-fold resistance [44]. Whereas HaABCC2, HaABCC3, and HaCad probably act as toxin receptors in *H*. *armigera*, HaTSPAN1 does not, because a strain with *HaTSPAN1* knocked out was completely susceptible [44]. The nature of the Cry1Ac-HaTSPAN1 interaction and the temporal sequence of interactions between Cry1Ac and the four aforementioned midgut proteins affecting its toxicity (HaABCC2, HaABCC3, HaCad and HaTSPAN1) remain to be determined.

It is useful to compare the results here with CRISPR-mediated knockouts of *HaABCC2*, *HaABCC3*, or both with previously reported resistance genetically linked with *HaABCC2* in the LF60 strain of *H*. *armigera* that was selected conventionally by exposing larvae to Cry1Ac in the laboratory [33]. In LF60, mis-splicing of *HaABCC2* introduces a stop codon yielding a truncated HaABCC2 protein [33]. Consistent with the results here, resistance to Cry1Ac was recessively inherited in LF60. However, relative to its unselected, susceptible parent strain (LF), resistance to activated Cry1Ac toxin was 1100-fold in LF60, which is greater than the 4-fold resistance in C2-KO seen here–even though both strains are not expected to produce functional HaABCC2 protein. The difference in results between studies could reflect differences between the strains studied, the experimental methods, or both. Although we used Cry1Ac produced by transgenic *E*. *coli* and the previous study used Cry1Ac produced by the HD73 strain of *B*. *thuringiensis* [33], this minor difference is unlikely to be responsible for the resistance to activated Cry1Ac toxin of only 4-fold in C2-KO versus 1100-fold in LF60. For LF60, the genetic linkage of resistance to Cry1Ac with mutant *HaABCC2* does not exclude the possibility of mutations in *HaABCC3* or other nearby genes that were not examined. Thus, one potential explanation is that, in addition to mis-splicing of *HaABCC2*, LF60 had a mutation in *HaABCC3* or another nearby gene that caused the 1100-fold resistance in concert with its *HaABCC2* mutation.

Similar to the results here where >15,000-fold resistance to Cry1Ac in C2/3-KO did not affect susceptibility to the ryanoid insecticide chlorantraniliprole, resistance of LF60 to Cry1Ac did not affect susceptibility to endosulfan (a cyclodiene), phoxim (an organophosphate), or cyhalothrin (a pyrethroid, [46]). However, susceptibility to the bacterially derived insecticides abamectin and spinetoram was unaffected in C2/3-KO here, but increased significantly in LF60 [46]. Similar to the explanation above, we cannot exclude the possibility that the negative cross-resistance in LF60 was caused by one or more mutations other than the *HaABCC2* mutation.

The results here showing that mutations in ABCC2 alone caused only about 4-fold resistance to Cry1Ac differ from previous studies of *H*. *armigera* and seven other lepidopteran species, where authors concluded that higher levels of resistance to Cry1Ab, Cry1Ac, Cry1Ca, or Cry1Fa is associated with alterations of ABCC2 [30–32, 34–40]. In most of these cases, as noted above for the previous work with *H*. *armigera* [33], we cannot exclude the hypothesis that in addition to mutations affecting ABCC2, mutations affected either the gene encoding ABCC3 or another nearby gene. However, in *Plutella xylostella*, resistance to Cry1Ac caused by CRISPR-mediated gene editing was 724-fold for knocking out *PxABCC2* alone and 423-fold for knocking out *PxABCC3* alone [36]. RNAi suppression of expression of either of these genes also significantly reduced susceptibility to Cry1Ac in *P*. *xylostella* [35]. Similarly, in *Spodoptera exigua*, RNAi suppression of either *SeABCC2* or *SeABCC3* alone significantly reduced susceptibility to Cry1Ac and Cry1Ca [34]. In *Spodoptera frugiperda*, knocking out *SfABCC2* alone caused 118-fold resistance to Cry1F [40]. These results imply that ABCC2 and ABCC3 are not functionally redundant in the toxic pathways of the Cry1 proteins evaluated in *P*. *xylostella*, *S*. *exigua*, and *S*. *frugiperda*.

Field-evolved resistance is more likely if one mutation is sufficient to substantially decrease susceptibility than if mutations in two different genes are required, as reported here for *HaABCC2* and *HaABCC3* and resistance to Cry1Ac. In a related example, mutations in *Trichoplusia ni* affecting either *TnABCC2* or *TnCad* alone caused less than 8-fold resistance to the genetically modified Bt toxin Cry1Ac-A01s, whereas knocking out both yielded 3800-fold resistance [48].

Although field-evolved resistance of *Pectinophora gossypiella* to Cry2Ab in India is associated with mutations affecting ABCA2 [24] and many cases of laboratory-selected resistance to Cry1 toxins are associated with mutations affecting ABCC2 [30,31,34–37,40], we know of only two species with field-evolved resistance where a mutation affecting ABCC2 is putatively implicated. In the GLEN-Cry1Ac-BCS strain of *T*. *ni* isolated from a greenhouse population in western Canada that evolved resistance to sprays containing a mixture of Bt toxins, resistance to Cry1Ac is genetically linked with *TnABCC2*, but the specific mutation has not been identified [31]. In *S*. *frugiperda* resistance to Cry1Fa in Puerto Rico, a mutant allele (called *SfABCC2mut* or R_1_) isolated from field populations harbors a 2-bp insertion in *SfABCC2*, which yields a premature stop codon and is associated with recessive resistance to Cry1Fa and cross-resistance to Cry1A.105 [38,39]. However, in 2007, soon after field failures were reported and resistance to Cry1Fa was confirmed with bioassays [49], the frequency of R_1_ was 0 and 0.014 at the two sites studied in Puerto Rico (n = 48 and 145, respectively, [38]). Thus, the frequency of R_1_ was too low in 2007 to account for the practical resistance observed then. Paradoxically, R_1_ was more common in Puerto Rico from 2009 to 2017 [38,39], after Cry1Fa-producing corn was reportedly withdrawn from the market there [49]. This suggests that R_1_ was favored by other types of Bt corn planted after 2007, such as multi-toxin corn producing Cry1Fa, Cry1A.105, or related toxins. Nonetheless, the frequency of R_1_R_1_ homozygotes was less than 0.50 in five of the six samples from 2009 to 2017 (Table S1 of [38], [39]). R_1_ was not detected in Cry1Fa-resistant populations in Florida, the Dominican Republic, or Brazil; and a second resistance allele (R_2_) of *SfABCC2* was isolated from the field in Puerto Rico, but its frequency was not reported [38,39]. A recent study identified many mutations in *SfABCC2* associated with field-selected resistance to Cry1Fa in Brazil [50]. As far as we know, mutations in *ABCC3* were not evaluated in the cases of field-evolved resistance in *T*. *ni* and *S*. *frugiperda* cited above. To better understand the practical impact of *ABCC2* and *ABCC3* resistance mutations, it will be useful to track their frequency in parallel with bioassays in field populations of *H*. *armigera* and other pests.

## Materials and methods

### Insect strains and rearing

We used four strains of *H*. *armigera*: the susceptible strain SCD, and three knockout strains generated from SCD in this study (C2-KO, C3-KO, and C2/3-KO). SCD was started with insects collected from Côte D’Ivoire (Ivory Coast, Africa) in the 1970s [51] and had been maintained in the laboratory without exposure to insecticides or Bt toxins for more than 30 years. C2-KO, C3-KO, and C2/3-KO had knockouts of *HaABCC2*, *HaABCC3*, or both, respectively, as detailed below.

All larvae were reared on a diet based on wheat germ and soybean powder at 26 ± 1 ^o^C, 60 ± 10% relative humidity and a photoperiod of 16 h light: 8 h dark. A 10% sugar solution was supplied for adults.

### Cas9 protein and sgRNAs

TrueCut^TM^ Cas9 Protein v2 was purchased from Thermo Fisher (Shanghai, China). The sgRNA target sequences were selected according to the principle of 5’-GN_19_NGG-3’. Four sgRNAs were used in this study (C2-sgRNA1 targeting at exon 4 of *HaABCC2*: GTGGATCGTGCCTATCCAAGTGG; C2-sgRNA2 targeting at exon 24 of *HaABCC2*: GGTCATGGACCAGGGCGAGGTGG; C3-sgRNA1 targeting at exon 4 of *HaABCC3*: GTGGCCGCGGTCATATACCTCGG; C3-sgRNA2 targeting at exon 25 of *HaABCC3*: GGTGAGCAGCGGCGAGATCGCGG; PAM sequences were underlined) (Fig 1A). The template DNA was synthesized with PCR-based fusion of two oligonucleotides: the specific oligonucleotide encoding T7 polymerase-binding site and the sgRNA target sequences (5’-GAAATTAATACGACTCACTATAGN_19_GTTTTAGAGCTAGAAATAGC-3’) and the universal oligonucleotide encoding the remaining sgRNA sequences (5’-AAAAGCACCGACTCGGTGCCACTTTTTCAAGTTGATAACGGACTAGCCTTATTTTAACTTGCTATTTCTAGCTCTAAAAC-3’). The fusion PCR reaction system and purification of PCR products were the same as reported by Wang et al. [47]. The sgRNAs were synthesized by in vitro transcription utilizing the GeneArt^TM^ Precision gRNA Synthesis Kit (Thermo Fisher Scientific, Lithuania) according to the manufacturer’s instruction.

### Embryo microinjection

Microinjection was done as reported previously [47]. Briefly, fertilized eggs laid within 2 hours were washed off from the gauzes in 1% sodium hypochlorite solution and rinsed with distilled water for three times, which was followed by suction filtration. Next the eggs were lined up on double-sided adhesive tape attached to a microscope slide. Each embryo was injected with approximately 1 nl of solution containing sgRNA and Cas9 protein. The injected eggs were incubated at 26 ± 1 ^o^C until hatching.

The C2-sgRNA1 and C2-sgRNA2 were co-injected to knock out the most of the full length of the genomic sequence of *HaABCC2*, while C3-sgRNA1 and C3-sgRNA2 were designed to delete a 22.8-kb fragment of *HaABCC3* (Fig 1B). All of the sgRNAs were effective at the final concentration of 500 ng/μl with Cas9 protein (200 ng/μl).

### Identification of mutation types induced by CRISPR/Cas9

Direct sequencing based on PCR products was conducted by TsingKe (Nanjing, China) to detect the mutation types on different target sites. Table 2 lists the primers. We used primer pair C2-tar1-F/C2-tar2-R to detect the 7.36-kb fragment knockout of *HaABCC2*, and C3-tar1-F/C3-tar2-R to detect the 22.8-kb fragment knockout of *HaABCC3*. The indel (insertion or deletion) mutation types were analyzed using a previously reported method [52].

### Bt toxins and insecticides

We purchased Cry1Aa, Cry1Ab and Cry1Ac activated toxins from Dr. Marianne Pusztai-Carey (Case Western Reserve University, USA). Abamectin (2% EC) and chlorantraniliprole (5% EC) were supplied by Guangdong Academy of Agricultural Sciences, Guangzhou, China. Spinetoram (6% SC) was purchased from Dow AgroSciences Ltd, USA.

### Bioassays

We used diet overlay bioassays [51] to determine susceptibility to Bt toxins and insecticides. We prepared the desired concentrations of Bt toxins and insecticides by diluting stock suspensions with a 0.01 M, pH 7.4 phosphate buffer solution (PBS). Artificial diet (1200 μl) was dispensed into each well (surface area = 2 cm^2^) of a 24-well plate. After the diet cooled and solidified, 100 μl of the dilution containing the desired concentration of Bt toxin or insecticide was applied evenly to the diet surface in each well. After the wells dried at room temperature, we put in each well a single unfed neonate for Bt toxins or second instar for insecticides. We tested 48 larvae at each concentration. Mortality was recorded after 7 days for Bt toxins or 3 days for insecticides. When mortality was scored, larvae were considered dead if they were dead or weighed less than 5 mg for Bt toxins or could not move normally for insecticides.

To determine the LC_50_ (concentration of a Bt toxin or insecticide killing 50% of larvae), we tested a series of concentrations including untreated diet as a control. Control mortality ranged from 0 to 2% (mean = 0.5%). The LC_50_ (concentration of a Bt toxin or insecticide killing 50% of larvae) and the 95% fiducial limits of the LC_50_ for each strain were calculated with probit analysis of the mortality data using PoloPlus [53]. Two LC_50_ values were considered significantly different if their 95% fiducial limits did not overlap. We calculated the resistance ratio for each toxin and insecticide tested as the LC_50_ for a strain divided by the LC_50_ for SCD. We also used Fisher’s exact test to determine if mortality at a diagnostic concentration of Cry1Ac (0.05 μg Cry1Ac per cm^2^) for each of three knockout strains differed significantly from mortality for SCD (n = 96 larvae per strain).

### Inheritance of resistance to Cry1Ac

To evaluate inheritance of resistance to Cry1Ac, we made all 12 possible interstrain reciprocal crosses (10 males X 10 females of each strain for each cross) between the SCD, C2-KO, C3-KO and C2/3-KO strains. In bioassays at 0.05 μg Cry1Ac per cm^2^ diet, we tested F_1_ offspring (48 larvae) from each of the 12 reciprocal crosses. To evaluate sex linkage and maternal effects, we used Fisher’s exact test to determine if differences occurred between the two reciprocal crosses for each interstrain cross (e.g., female SCD X male C2-KO vs. female C2-KO X male SCD). We also used Fisher’s exact test to determine if mortality for F_1_ progeny from each of the six interstrain crosses (n = 96 larvae per cross) differed significantly from mortality for SCD and if mortality differed significantly between the F_1_ progeny from C2/3-KO X C2-KO and C2/3-KO X C3-KO.
